# Degradation by water vapor of hydrogenated amorphous silicon oxynitride films grown at low temperature

**DOI:** 10.1038/s41598-017-14291-2

**Published:** 2017-10-26

**Authors:** Hyung-Ik Lee, Jong-Bong Park, Wenxu Xianyu, Kihong Kim, Jae Gwan Chung, Yong Koo Kyoung, Sunjung Byun, Woo Young Yang, Yong Young Park, Seong Min Kim, Eunae Cho, Jai Kwang Shin

**Affiliations:** Samsung Advanced Institute of Technology, 130, Samsung-ro, Yeongtong-gu, Suwon-si, Gyeonggi-do, 16678 Korea

## Abstract

We report on the degradation process by water vapor of hydrogenated amorphous silicon oxynitride (SiON:H) films deposited by plasma-enhanced chemical vapor deposition at low temperature. The stability of the films was investigated as a function of the oxygen content and deposition temperature. Degradation by defects such as pinholes was not observed with transmission electron microscopy. However, we observed that SiON:H film degrades by reacting with water vapor through only interstitial paths and nano-defects. To monitor the degradation process, the atomic composition, mass density, and fully oxidized thickness were measured by using high-resolution Rutherford backscattering spectroscopy and X-ray reflectometry. The film rapidly degraded above an oxygen composition of ~27 at%, below a deposition temperature of ~150 °C, and below an mass density of ~2.15 g/cm^3^. This trend can be explained by the extents of porosity and percolation channel based on the ring model of the network structure. In the case of a high oxygen composition or low temperature, the SiON:H film becomes more porous because the film consists of network channels of rings with a low energy barrier.

## Introduction

Most organic conducting polymers and chemically reactive electrodes undergo degradation when exposed to water or oxygen. Therefore, device encapsulation using barrier materials with low permeability for water vapor and oxygen is inevitably required for realizing organic devices with a long lifetime^1–4^. The most commonly used method is encapsulation by glass and a metal lid. However, because these rigid materials are incompatible with flexible organic devices, a technique called thin film encapsulation (TFE) has been introduced. This approach has led to research on various methods for depositing films such as SiN, SiO, SiON, and AlO^5–8^.

Silicon oxynitride is particularly attractive for photoelectric applications because of high transmittance and adjustable refractive index from 1.45 (SiO_2_) to 2.01 (Si_3_N_4_) simply by changing the ratio of O/N. However, when such a film is deposited as a single layer at low temperature, it is usually insufficient to protect the organic device from water or oxygen. Although the TFE performance can be significantly improved by using multilayer films^7,8^, understanding the fundamental degradation mechanism of a single layer by water vapor or oxygen is very important for developing a high-quality TFE film.

Usually, the Ca corrosion test is used to evaluate the protection by TFE from water vapor and oxygen^2,9^. Because Ca is not only reactive with water and oxygen but also conductive, the water vapor transmission rate (WVTR) can be measured with high accuracy. It can be measured to as low as 10^−6^ g/m^2^/day, which is required for a long lifetime in organic devices. Although the Ca corrosion test is good for evaluating TFE, it is limited in helping understand the TFE degradation process by water. The pressure cooker test (PCT) is another method for directly investigating film stability against water.

In general, the permeation of water through TFE is caused by nano-voids or defects such as pinholes^10–13^. These nano-voids or defects are more severe at low deposition temperatures. Therefore, characterization of properties such as the atomic concentration, mass density, oxidation, and chemical state is necessary for investigating the degradation process. The structure of the SiON film can be estimated from the atomic concentration and chemical bond state. In particular, a SiON film deposited by plasma-enhanced chemical vapor deposition (PECVD) using SiH_4_, NH_3_ and N_2_O gas contains many hydrogen atoms. They also cause nano-structural changes according to atomic concentration or deposition temperature. The mass density is the most essential parameter because the TFE stability can be estimated from the density of only as-deposited samples without a permeation test. The SiON:H film that forms as a result of the PCT is fully oxidized. Therefore, the TFE degradation mechanism due to water can be considered to be an oxidation process, and the extent of degradation can be estimated from the fully oxidized thickness after the PCT.

In this paper, we report on the degradation process of a SiON:H film caused by water vapor by presenting an analysis of the characterization results such as atomic concentration, mass density, chemical bond state, oxidation process, and oxidation thickness. The degradation of a SiON:H film deposited by PECVD at low temperature was investigated as a function of the oxygen concentration and deposition temperature. All of the samples were subjected to the PCT and characterized by using analytical tools. In order to understand the origin of the degradation, the measured results were also analyzed according to the porosity based on the network structure model. The results showed that a dense film is required for achieving TFE with a high energy barrier.

## Experiment

Hydrogenated amorphous silicon oxynitride films were deposited by PECVD on (001) p-type Si substrates in a Novellus C2 reactor. The chamber pressure, rf power, and rf frequency were 2 Torr, 200 W, and 13.56 MHz, respectively. Two set of films with various levels of oxygen content and grown at various deposition temperatures were prepared. All of the films were deposited at the same constant gas flow of 10 sccm for both SiH_4_ and NH_3_. Only the N_2_O gas flow or deposition temperature was varied. One set was deposited with various levels of oxygen content and N_2_O flows of 0–14 sccm, but the deposition temperature was maintained at 160 °C. The other set was carried out at a temperature range of 100–300 °C and a constant N_2_O flow of 6 sccm. The degradation of SiON:H by water vapor occurred under accelerated test (i.e., PCT) conditions of 121 °C, 2 atm, and a relative humidity of 100%.

The degradation trend and water vapor diffusion were analyzed by using transmission electron microscopy (TEM) images and time-of-flight secondary ion mass spectroscopy (ToF-SIMS) spectra. In particular, the degradation trend was clearly investigated by mapping two-dimensional oxidation regions in the depth and lateral directions by using energy dispersive spectroscopy (EDS) in TEM. For the ToF-SIMS analysis, a Bi^+^ ion beam of 25 keV was used to generate secondary ions, and a Cs^+^ ion beam of 2 keV was used for sputtering.

The thickness of fully oxidized layers after PCT and atomic concentrations of Si, O, and N were measured by using high-resolution Rutherford backscattering spectroscopy (HR-RBS). The HR-RBS spectra were obtained from a He^+^ ion beam of 450 keV by using HRBS-V500 of Kobe Steel, Ltd. The hydrogen was measured by elastic recoil detection analysis (ERDA) using the same system as for HR-RBS but with a N^+^ ion beam of 500 keV. X-ray reflectometry (XRR) was used to measure the film thickness and mass density. Because the areal density can also be calculated from HR-RBS spectra, the mass density was determined by comparing the RBS density with that of XRR. Fourier transform infrared spectroscopy (FTIR) and X-ray photoelectron spectroscopy (XPS) were used to analyze the chemical bonds and depth profiling of the samples, respectively.

## Results and Discussion

**Figure 1:**
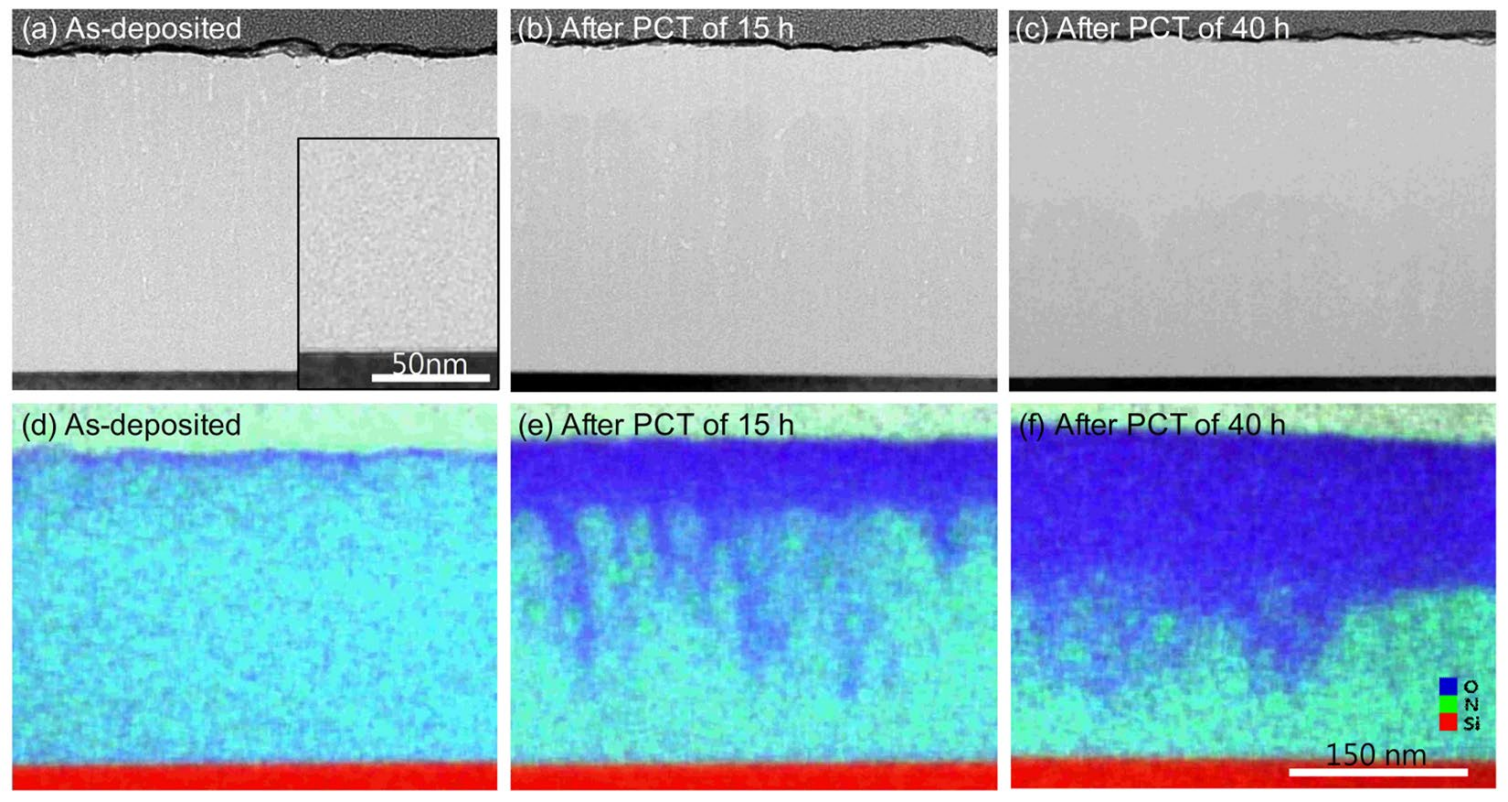
(**a**–**c**) TEM images and (**d**–**f**) TEM-EDS elemental mapping images after PCT of 0, 15, and 40 h for a SiON:H film, respectively. As-deposited sample was deposited on Si substrate at 160 °C and a N_2_O flow of 14 sccm. The blue, green and red parts in (**d**,**e**) and (**f**) represent SiO_2_:H, SiON:H and Si substrate regions, respectively. The scales of all the images are the same as shown in Fig. 1(f).

**Figure 2:**
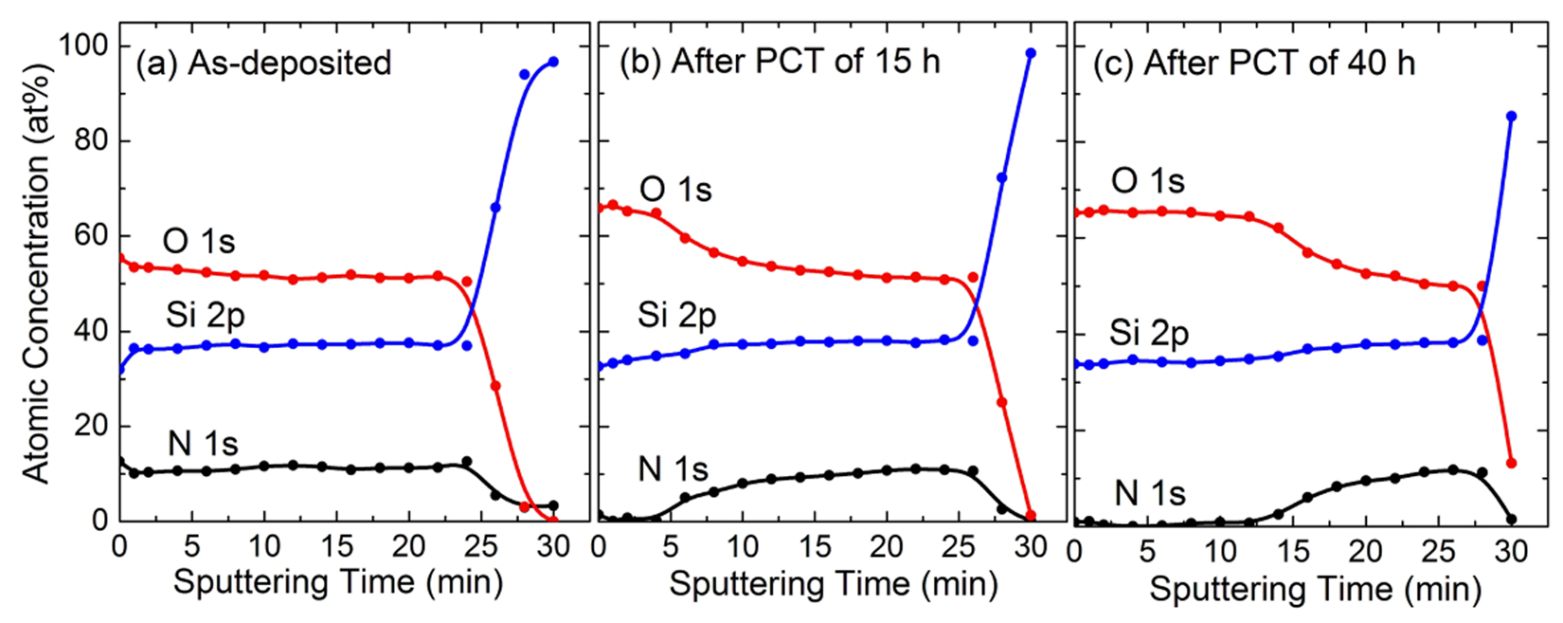
(**a**–**c**) XPS sputter depth profiles corresponding to PCT of 0, 15, and 40 h in Fig. 1(a–c), respectively. The films were etched with Ar^+^ ion beam of 1.0 keV.

In general, a SiON film deposited at low temperature oxidizes very easily when exposed to water vapor. In terms of bond energy, the strength of the Si–O bond (799.6 kJ/mol) is stronger than those of the Si–Si (325.0 kJ/mol), Si–N (470.0 kJ/mol), N–H (339.0 kJ/mol), O–H (427.6 kJ/mol), and Si–H (299.2 kJ/mol) bonds^14^. If PCT is performed over a long time, all of the SiON layers will eventually convert to a SiO_2_:H film. Therefore, the degradation process by water vapor can be studied by directly measuring the fully oxidized domains. Figure 1(a–c) show TEM images after PCT of 0, 15, and 40 h for SiON:H, deposited at 160 °C and a N_2_O flow of 14 sccm. The Si, O, N, and H concentrations of as-deposited sample obtained from HR-RBS and ERDA were 28.3, 38.5, 15.3, and 17.9 at%, respectively. Figure 1(d–f) show TEM-EDS elemental mapping images corresponding to Fig. 1(a–c), respectively. The blue, green, and red parts of Fig. 1(d–f) indicate fully oxidized SiO_2_:H, silicon oxynitride, and silicon substrate regions, respectively. XPS sputter depth profiles of Si 2p, O 1 s, and N 1 s signals corresponding to PCT time of 0, 15, and 40 h in Fig. 1(a–c) show in Fig. 2(a–c), respectively. The depth profiles were obtained with Ar^+^ ion beam of 1.0 keV. The concentration profiles for as-deposited sample were uniform. In the surface region after PCT of 15 and 40 h, the signal of N 1s was not observed and the composition ratios of Si and O were SiO_2_.

Figure 1(d–f) visualize the progression of water vapor diffusion over the time of the PCT. The TEM-EDS image for as-deposited sample of Fig. 1(d) shows that there was a little surface oxidation due to ambient exposure. This result can be confirmed from the XPS depth profile of Fig. 2(a). As the PCT time increased, the SiON:H film become more thick oxidized. In particular, Fig. 1(e) shows that there were two different oxidized regions. That is, the SiON:H film was oxidized by water vapor diffusing through different two paths. For one diffusion path, the water vapor gradually penetrated from the surface downward over time. After PCT time of 15 h, all layers within a ~60 nm thickness from the surface were completely oxidized. For the other diffusion path, water vapor propagated from the surface to a distant location, but only narrow and long regions were oxidized. This type of diffusion has usually been attributed to pinhole-related defects^10^. However, no pinhole defects in the interface between SiON:H and Si substrate were visible in the magnified TEM image inserted in Fig. 1(a). In TEM image of Fig. 1(a), several nano-voids were only observed from the surface to tens of nano-meters. Because diffusion of water vapor into these nano-voids was faster than first case, narrow and long pattern of oxidation was formed. After PCT of 40 h, the oxidized region was relatively uniform because nano-voids decreased toward the interface.

**Figure 3:**
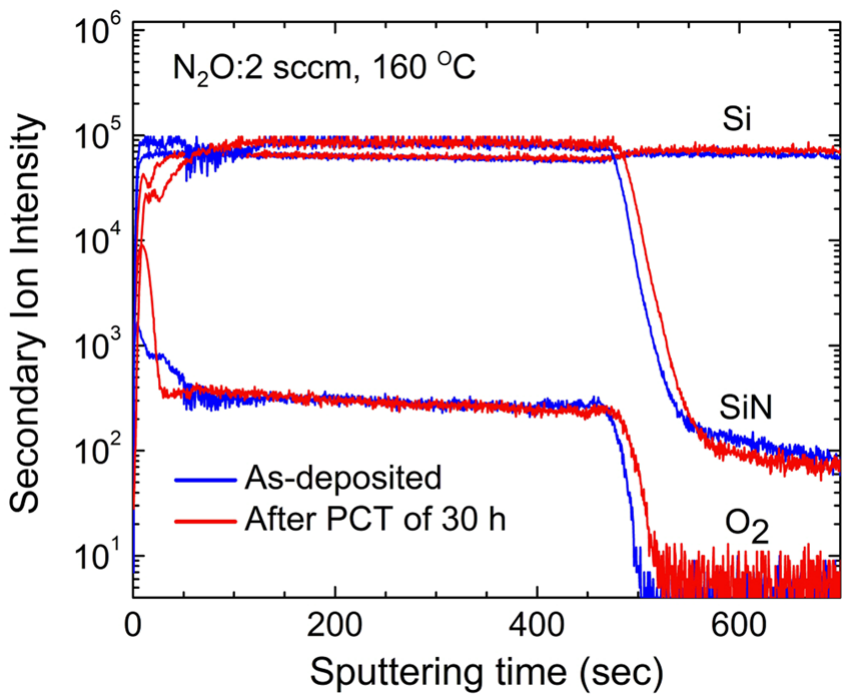
SIMS depth profiles of SiON:H grown at a deposition temperature of 160 °C and N_2_O flow of 2 sccm. The blue lines are profiles before PCT, and the red lines are profiles after PCT of 30 h.

Whether or not the film oxidizes through pinhole defects can also be confirmed by SIMS, which can measure depth profiles very sensitively. Figure 3 shows ToF-SIMS depth profiles before and after a PCT of 30 h for the SiON:H film with high nitrogen content. The sample was deposited at 160 °C with a N_2_O flow of 2 sccm, and the thickness was 250 nm. The concentrations of Si, O, N, and H atoms were 27.0, 9.8, 37.3, and 25.9 at%, respectively. The O_2_ profile after the PCT of 30 h shows that fully oxidized layers appeared up to a depth of ~7 nm (until 30 s of sputtering). However, after sputtering for 30 s, there was almost no difference between the O_2_ profiles before and after the PCT. If there were pinhole defects in the film, a small profile difference would appear at deep ranges because a film with high nitrogen content is more protective against water vapor.

**Figure 4:**
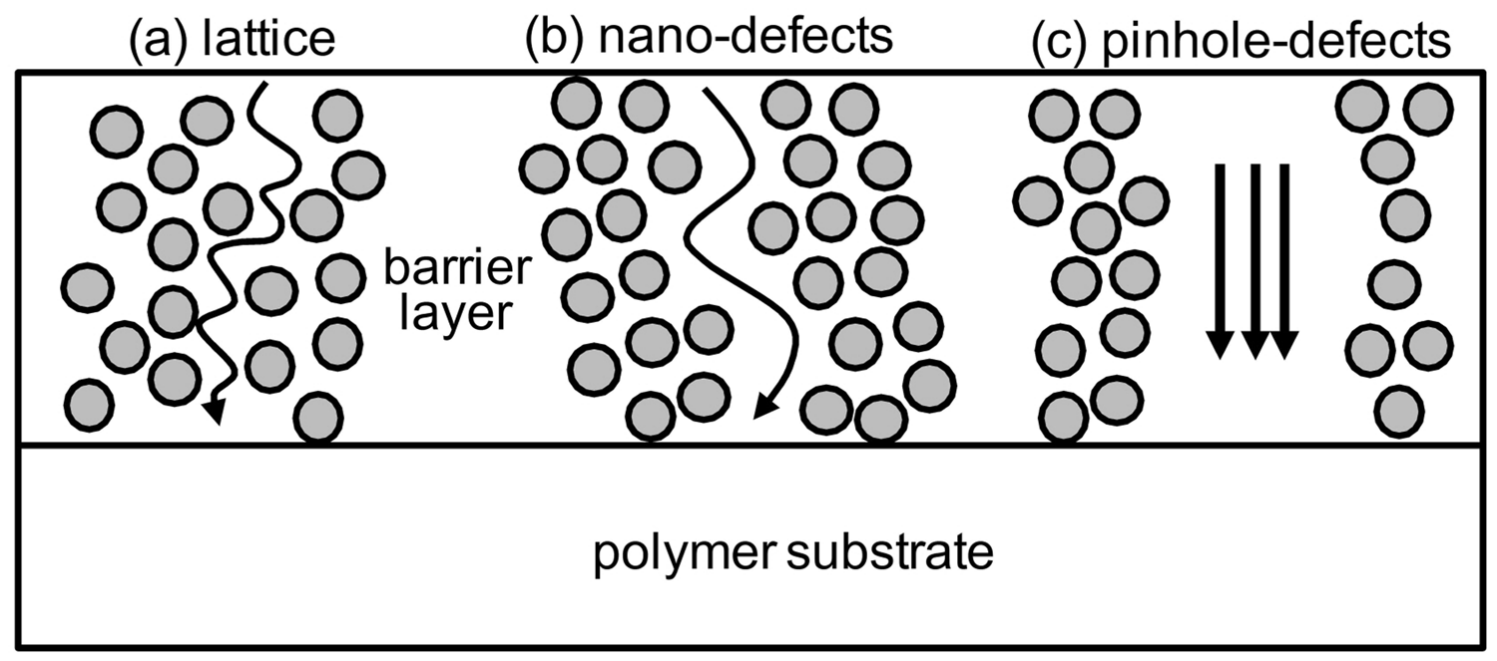
Schematic diagram showing permeation pathways of water vapor proposed by Roberts *et al*.^13^.

This type of diffusion was shown in schematic diagram of Fig. 4 for (a) lattice, (b) nono-defects, and (c) pinhole-defects, proposed by Roberts *et al*.^13^. In the present study, only the diffusion paths by (a) lattice or interstitial and (b) nano-defects were observed. Usually diffusion by interstitial path does not occur well, but it dominates the diffusion path because the sample was deposited at low temperature in the present study. The long-range diffusion by nano-defects observed with TEM is considered to be from the water vapor passing through voids in the size range of sub-nanometer to several nanometers that were produced during film fabrication. Therefore, the measurement of the completely oxidized thickness caused by interstitial path is enough to observe the degradation trend of SiON:H as shown in the TEM and SIMS results. The trend of the fully oxidized thickness will be different according to the oxygen content or deposition temperature.

**Figure 5:**
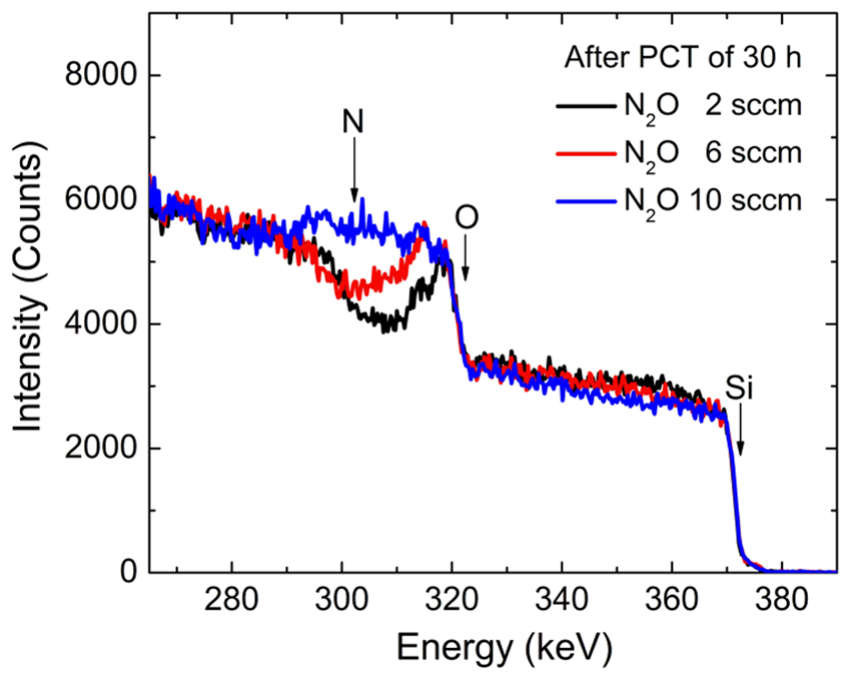
HR-RBS spectra of SiON:H films after PCT of 30 h for N_2_O gas flows of 2, 6 and 10 sccm as given in Table 1.

We first investigated SiON:H films with various levels of oxygen and nitrogen contents and deposited at a fixed temperature of 160 °C. Table 1 presents the mass densities and atomic concentrations of Si, O, N, and H before the PCT. All of the samples were subjected to a PCT of 30 h. The last column of Table 1 indicates the thicknesses of the fully oxidized SiO_2_:H layers after the PCT, measured by HR-RBS spectra. Figure 5 shows the related HR-RBS spectra for N_2_O flows of 2, 6, and 10 sccm. As the N_2_O gas flow increased, the mass densities and atomic concentrations of N and H decreased, but the O concentration and fully oxidized thickness increased. The concentration of Si atoms stayed at approximately ~30 at%. Therefore, the degradation of SiON:H is closely related to the increase in O atoms.

**Table 1:** Mass density and atomic concentration of SiON:H films as a function of the N_2_O gas flow. The last column represents the thicknesses of fully oxidized SiO_2_:H after PCT of 30 h. The samples were deposited at 160 °C and 10 sccm for both SiH_4_ and NH_3_.

N_2_O sccm	Density g/cm^3^	Si at%	O at%	N at%	H at%	Oxidation nm
0	2.25	27.9	0.0	45.2	26.8	5
2	2.22	27.0	9.8	37.3	25.9	7
4	2.18	29.1	16.5	32.2	22.2	8
6	2.17	28.7	20.5	28.8	22.0	10
8	2.15	29.7	26.9	24.5	18.9	18
10	2.13	29.1	32.3	20.8	17.9	30

The degradation trend for the oxygen content can be explained in terms of the activation energy. For the diffusion coefficient, the Arrhenius law *D* = *D*_o_exp(−*E*_*a*_/*kT*) usually holds. Here, *D*_o_ is the diffusion constant that can be estimated by using the Einstein relation, *E*_*a*_ is the activation energy, *k* is the Boltzmann constant, and *T* is the deposition temperature^13,15^. The activation energy for Si_3_N_4_ (2.1 eV) is higher than that for SiO_2_ (0.5 eV)^16^. Therefore, the diffusion coefficient of water vapor will increase with increasing oxygen content. The activation energy represents the barrier to interstitial diffusion shown in Fig. 4(a). This means that SiON:H films with higher oxygen content than SiON:H or SiN:H should possess a more porous structure. The mass densities presented in Table 1, which describe the porous extent, show this trend very well. However, the density linearly decreased with increasing oxygen content, while the fully oxidized thickness rapidly increased from a density of ~2.15 g/cm^3^ at an oxygen concentration of ~27 at%. This is a critical phenomenon that occurs when many percolation channels are formed by these porous structures. The diffusivity of water vapor can be estimated from the random walk model^17^ *L* ^2^ = 4*Dt*, where *L*, *D*, and *t* are the fully oxidized thickness, diffusion coefficient, and PCT time, respectively. This model calculates a diffusion coefficient of ~2 × 10^−18^ cm^2^/s for a SiON:H film with an oxygen content of ~20 at%. However, the diffusion coefficient for films with high oxygen content is overestimated because the film is changed by thick SiO_2_:H layers with a higher diffusion coefficient during a PCT^17,18^.

**Figure 6:**
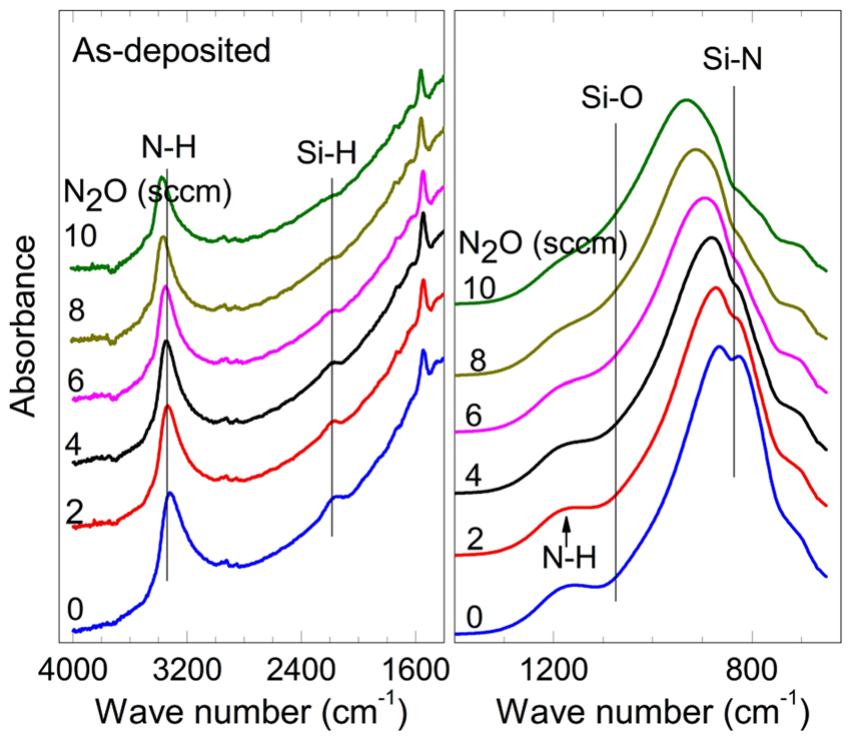
FTIR spectra of SiON:H films before PCT at various N_2_O gas flows.

In terms of nanostructures, the reason why the porosity varies with the oxygen concentration can also be explained by the difference between the chemical bonds of the films. Amorphous SiON:H films can be characterized by a structure of networks with rings of various member rings^19–21^. The number of members in a ring for amorphous SiO_2_, which is defined as the number of Si atoms, can range from three to nine, and six-member rings occur most frequently. Bakos *et al*. calculated that the activation energy for water molecules in SiO_2_ is 0.8–0.9 eV for six- and seven-member rings, but four- and five-member rings have a higher barrier of 1.8–2.2 eV^21^. Hence, water vapor can penetrate across rings with members of large sizes, which can constitute a diffusion channel. The number of members in a ring can have different statistical distributions depending on the chemical bond configuration. Because the ratios of [(O+N)/Si] in Table 1 are close to 2, SiON:H had a structure similar to SiO_2_. Hence, the Si atoms of SiON were mainly bonded with oxygen or nitrogen as also shown by the FTIR results of Fig. 6 ^22^. Because the coordination number of nitrogen (3) is larger than that of oxygen (2), the network structure comprising Si–N–Si bonds has a statistically smaller ring size than that comprising Si–O–Si bonds. Therefore, a SiN film is more resistive against water vapor than a SiO_2_ film.

The porosity also depends on the hydrogen bonds as well as the Si–N–Si and Si–O–Si bonds. In particular, if a film is composed of many Si–N–H or Si–O–H bonds, the porous feature of the film will statistically increase because the coordination numbers of nitrogen and oxygen excluding hydrogen bonds are each reduced by one. For hydrogen atoms, most bonds in the SiON:H film were N–H, as shown by the FT-IR results of Fig. 6. In fact, the ERDA results shows that the hydrogen atoms in the SiO_2_:H film that formed from SiON:H after the PCT decreased remarkably from 18–27 at% before the PCT to ~9 at% after PCT. This is because the Si–N–H bond was replaced with more stable Si–O–Si or Si–O–H bonds by the reaction with water molecules. Because of the reaction results, hydrogen atoms desorbed from the surface as NH_3_ molecules, and only Si–H and O–H bonds remained in the film^16^.

The SiN:H sample without oxygen content, as presented in 0 sccm of Table 1, contained a high concentration of hydrogen atoms at 26.8 at%; this included at least 17.8 at% of N–H bonds^23^. Hence, it should be composed of porous structures, and the density was really only 2.25 g/cm^3^. However, the sample stability was excellent because sufficient diffusion channels for water vapor penetration did not form. If a considerable number of hydrogen atoms are contained in a SiN:H film, it can degrade easily. Similarly, if the sample gradually changes from SiN:H to SiON:H with higher levels of oxygen content so that the Si–O–Si bonds increase, the sample becomes more porous. Eventually, a critical oxygen concentration is reached where many percolation channels are formed so that the sample rapidly degrades by the reaction with water vapor^16^.

The SiON:H film became more porous with increasing oxygen and hydrogen atoms, but the fundamental cause of the porous structure even for the SiN:H film is the film growth at low temperature^24^. In order to investigate the degradation process for the deposition temperature, SiON:H samples were grown at a range of 100–300 °C and a constant N_2_O flow of 6 sccm. Table 2 presents not only the mass density and atomic concentrations before the PCT but also the fully oxidized SiO_2_:H thickness after a PCT of 30 h. For the 100 °C sample, because all of the SiON:H layers up to a depth of ~100 nm were fully oxidized after the PCT, its oxide thickness is not described in Table 2.

**Table 2:** Mass density and atomic concentration of SiON:H films as a function of the deposition temperature. The last column represents the thicknesses of fully oxidized SiO_2_ after PCT of 30 h. The samples were deposited at 6 sccm for N_2_O and 10 sccm for both SiH_4_ and NH_3_.

Temperature °C	Density g/cm^3^	Si at%	O at%	N at%	H at%	Oxidation nm
100	2.12	26.6	26.8	24.0	22.6	
150	2.17	27.6	24.1	26.0	22.3	13.0
200	2.25	29.3	17.2	31.2	22.3	5.5
250	2.30	29.8	15.3	33.6	21.3	3.5
300	2.47	31.2	13.6	35.1	20.1	3.5

As the deposition temperature increased, the atomic densities and concentrations of Si and N increased, but the concentrations of O and H decreased. Therefore, the porosity or density in this case depended on the atomic composition as well as the deposition temperature. The samples can be denser at a higher deposition temperature because of the increase in nitrogen atoms and decrease in hydrogen atoms. However, if the samples with similar compositions in Tables 1 and 2 are compared, the densities only differ because of the deposition temperature. The compositions of the samples with a N_2_O flow of 4 sccm (Table 1) and 250 °C (Table 2) were similar, but the densities differed at 2.18 and 2.30 g/cm^3^, respectively. Namely, the sample deposited at a higher temperature became denser. In contrast, the samples deposited at a lower temperature became more porous. This can be observed by comparing the samples with a flow of 8 sccm (Table 1) and temperature of 100 °C (Table 2).

The change in porosity due to the deposition temperature can be explained as a mechanism of the film growth with the PECVD technique. In PECVD, the film is grown by adatoms forming by plasma and adsorbing onto the surface before migrating on the surface. At a low deposition temperature, the mobility of the adatoms is so low that the film grows almost randomly. Thus, a porous structure with large ring sizes is easily formed. The nano-voids in the TEM image of Fig. 1(a) can also be considered to be due to random growth^25^. As presented in Table 2, the fully oxidized thicknesses changed very dramatically when the deposition temperature was decreased from 150 °C to 100 °C, even though the difference in concentrations was small. This trend means that the film degradation with the PCT approached a critical temperature. The density at 150 °C was ~2.17 g/cm^3^, which is similar to the value in Table 1. The critical densities for different concentrations and deposition temperatures must have similar values because the degradation originates from a porous structure.

The water vapor diffused through percolation channels and nano-defects as discussed above reacts with SiON:H. As the reaction with water vapor progresses, Si-N bonds are gradually converted to Si-O bonds, eventually the SiON:H film becomes a fully oxidized film. The reaction process can be considered similar to the oxidation of Si_3_N_4_ film by water vapor (Si_3_N_4_ + 6 H_2_O → 3 SiO_2_ + 4 NH_3_)^16^ or the etching of Si_3_N_4_ film by HF^26,27^. As a result of reaction with water vapor, Si-N or Si-NH bonds are converted to Si-NH, Si-OH, or Si-NH_2_ bonds^16,24,28^. The Si-NH_2_ bond re-attacked by water vapor forms Si-OH bond and NH_3_ molecule (Si-NH_2_ + H_2_O → Si-OH + NH_3_), and the released NH_3_ molecule desorbs from the surface after diffusion in the surface direction. By repeating this process, the SiON:H film is gradually oxidized. Although the reaction speed varies depending on bond configuration of nitrogen and oxygen, the regions where water vapor is diffused are completely oxidized after a sufficiently long time.

The degraded SiO_2_:H regions formed in this way can play as another diffusion path for water vapor. In order to study the degradation of SiO_2_:H film formed by the reaction with water vapor, the density and composition were also investigated according to the PCT time for the SiON:H film deposited with N_2_O flow of 10 sccm as shown in Table 3. The density and atomic concentration after PCT of 30, 50, and 70 h in Table 3 indicates the values in the oxidized SiO_2_:H regions. The density and atomic composition after PCT were almost unchanged. The density was 2.0 g/cm^3^ and atomic concentration ratio of Si and O was SiO_2_. Therefore, once the SiON:H film was degraded to SiO_2_:H, no further degradation was observed. However, degraded SiO_2_:H regions acted as a diffusion channel for water vapor. Since the density in the SiO_2_:H region was lowered to 2.0 g/cm^3^, the water vapor in SiO_2_:H region diffuses faster than SiON:H regions.

**Table 3:** Mass density and atomic concentration of SiON:H films as a function of PCT time. The as-deposited film was deposited at 160 °C and 10 sccm for N_2_O. The density and composition after PCT indicates the values in the oxidized layers.

PCT time h	Density g/cm^3^	Si at%	O at%	N at%	H at%
0	2.10	29.6	33.0	21.1	16.3
30	2.00	30.1	61.8	—	8.1
50	2.00	29.3	62.2	—	8.2
70	2.00	29.0	63.0	—	8.0

**Figure 7:**
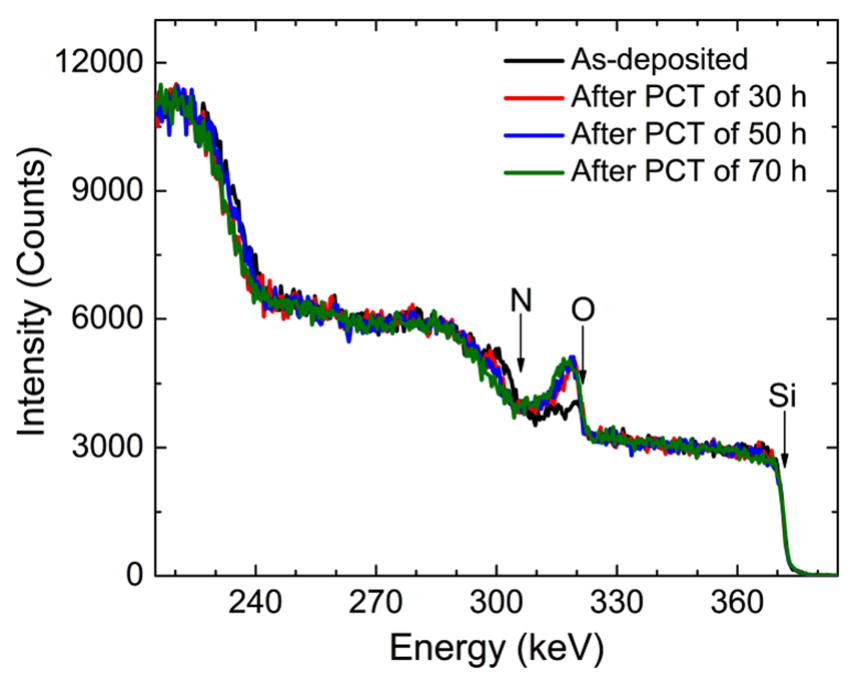
HR-RBS spectra of a bi-layer film, SiON:H (N_2_O:2 sccm, 30 nm) on SiON:H (N_2_O:10 sccm, 100 nm), after PCT of 0, 30, 50, 70 h.

Thus, in practical applications, it is necessary to block or reduce diffusion channels to realize a barrier film with excellent performance. The film fabricated by atomic layer deposition (ALD) rather than PE-CVD^2^ or at higher temperatures than at low temperatures^24^ are useful in reducing diffusion channels because they grows at a higher density. The multilayer film also blocks diffusion channels very well. Figure 7 shows HR-RBS spectra of a bi-layer film after PCT of 0, 30, 50, and 70 h. The first layer was a thin SiON:H film of 30 nm that was protective against diffusion of water vapor, grown by N_2_O gas of 2 sccm. Contrary, the second layer was a thick SiON:H film of 100 nm that diffusion of water vapor occurred very well, grown by N_2_O gas of 10 sccm. Although the PCT time was taken for a long time, the thickness of fully oxidized layers slightly increased and the water vapor was well blocked.

## Summary

The degradation by water vapor of hydrogenated amorphous silicon oxynitride film deposited by PECVD at low temperature was investigated as a function of the oxygen concentration and deposition temperature. The TEM and TEM-EDS observations showed no pinholes or grains, so the water vapor penetrated by diffusion through interstitial paths and nano-defects. Therefore, a porous structure formed with nano-voids of various sizes through which water vapor could penetrate. The relation between the porosity and water vapor penetration was experimentally confirmed by measurements of the density before a PCT and fully oxidized thickness after the PCT. The results showed that the SiON:H films became more porous with increasing oxygen concentration and decreasing deposition temperature, and the films rapidly degraded below a density of 2.15 g/cm^3^. This density corresponds to an oxygen concentration of ~27 at% and deposition temperature of ~150 °C.

These trends can be explained by a ring model with a network structure. Water vapor can diffuse through rings of large sizes with low activation energy. The ring size depends on the chemical bonding configuration. The Si–O–Si, Si–N–H, and Si–O–H bonds are fundamentally weak at protecting against water vapor because structures with large ring sizes are easy to form statistically. Although films with many oxygen or hydrogen atoms have porous characteristics, a denser film is possible when it is grown at a higher deposition temperature.
